# POSITIVE study: physical exercise program in non-operable lung cancer patients undergoing palliative treatment

**DOI:** 10.1186/s12885-016-2561-1

**Published:** 2016-07-19

**Authors:** Joachim Wiskemann, Simone Hummler, Christina Diepold, Melanie Keil, Ulrich Abel, Karen Steindorf, Philipp Beckhove, Cornelia M. Ulrich, Martin Steins, Michael Thomas

**Affiliations:** Working Group Exercise Oncology, Division of Medical Oncology, National Center for Tumor Diseases (NCT) and University Clinic Heidelberg, Heidelberg, Germany; National Center for Tumor Diseases (NCT) and German Cancer Research Center, Heidelberg, Germany; Clinic for Thoracic Diseases, Department of Oncology, Thoraxklinik am Universitätsklinikum, Heidelberg, Germany; Clinic for Thoracic Diseases, Department of Pneumology and Intensive Care Unit, Thoraxklinik am Universitätsklinikum Heidelberg, Heidelberg, Germany; Translational Lung Research Center Heidelberg (TLRC), Member of the German Center for Lung Research (DZL), Heidelberg, Germany; Immune Monitoring Unit (G808), National Center for Tumor Diseases (NCT) and German Cancer Research Center (DKFZ), Heidelberg, Germany; CCU Neuroimmunology and Brain Tumor Immunology, National Center for Tumor Diseases, (NCT) and German Cancer Research Center (DKFZ), Heidelberg, Germany; Huntsman Cancer Institute, Salt Lake City, Utah USA

**Keywords:** Lung cancer, Physical exercise, Quality of life, Fatigue, Care management phone calls, Palliative treatment

## Abstract

**Background:**

Patients with advanced stage non-small cell lung cancer (NSCLC) or small cell lung cancer (SCLC) often experience multidimensional impairments, affecting quality of life during their course of disease. In lung cancer patients with operable disease, several studies have shown that exercise has a positive impact on quality of life and physical functioning. There is limited evidence regarding efficacy for advanced lung cancer patients undergoing palliative treatment. Therefore, the POSITIVE study aims to evaluate the benefit of a 24-week exercise intervention during palliative treatment in a randomized controlled setting.

**Methods/design:**

The POSITIVE study is a randomized, controlled trial investigating the effects of a 24-week exercise intervention during palliative treatment on quality of life, physical performance and immune function in advanced, non-operable lung cancer patients. 250 patients will be recruited in the Clinic for Thoracic Diseases in Heidelberg, enrolment begun in November 2013. Main inclusion criterion is histologically confirmed NSCLC (stage IIIa, IIIb, IV) or SCLC (Limited Disease-SCLC, Extensive Disease-SCLC) not amenable to surgery. Patients are randomized into two groups. Both groups receive weekly care management phone calls (CMPCs) with the goal to assess symptoms and side effects. Additionally, one group receives a combined resistance and endurance training (3x/week). Primary endpoints are quality of life assessed by the Functional Assessment of Cancer Therapy for patients with lung cancer (FACT-L, subcategory Physical Well-Being) and General Fatigue measured by the Multidimensional Fatigue Inventory (MFI-20). Secondary endpoints are physical performance (maximal voluntary isometric contraction, 6-min walk distance), psychosocial (depression and anxiety) and immunological parameters and overall survival.

**Discussion:**

The aim of the POSITIVE trial is the evaluation of effects of a 24-week structured and guided exercise intervention during palliative treatment stages. Analysis of various outcomes (such as quality of life, physical performance, self-efficacy, psychosocial and immunological parameters) will contribute to a better understanding of the potential of exercise in advanced lung cancer patients. In contrast to other studies with advanced oncological patients the POSITIVE trial provides weekly phone calls to support patients both in the intervention and control group and to segregate the impact of physical activity on quality of life.

**Trial registration:**

ClinicalTrials.gov NCT02055508 (Date: December 12, 2013)

## Background

Worldwide, lung cancer is one of the most commonly diagnosed cancer types and also the leading cause of death in males [1]. Lung cancer incidence rates are highest in Europe and Northern America [2] and lung cancer belongs to the most aggressive human cancers with a 5 year survival rate of 10-15 % [3]. For patients with metastatic disease, 5 year overall survival is 2 % [4].

Advanced lung cancer patients often experience multidimensional impairments affecting quality of life during their course of disease [5]. Impairments result from symptoms of the disease and comorbidities on the one hand and side effects of treatment on the other hand. Moreover, socioeconomic problems arise and patients lose their social and professional integration. They are facing physical fragility and in many cases end-of-life situations [6]. A study in a representative sample of advanced cancer patients (*n* = 1630) in Denmark observed that lung cancer patients had exacerbated symptom burden (e.g. fatigue, dyspnea, pain, appetite loss), and presented with reduced physical and emotional function and quality of life, when compared with other cancer diagnoses like head and neck, gynecological, prostate, breast, gastrointestinal or bladder tumors [5]. Other investigations showed that patients with advanced lung cancer have specific problems such as coughing, shortness of breath, anorexia, and insomnia that need to be addressed [7–10].

Numerous studies have shown that physical activity positively affects quality of life, physical capacity and fatigue in cancer patients, irrespective of the tumor type [11–13]. Specifically for lung cancer patients, exercise studies in the pre-and post-operative setting showed an improvement in physical performance and cardiorespiratory fitness [14–18].

In contrast, studies investigating the effects of physical exercise in non-operable patients with advanced lung cancer are rare. The number of randomized controlled trials is limited and current knowledge is derived from feasibility and observational studies [19–23].

Previous investigations from our team with 39 lung cancer patients (POSITIVE study, Part I) assessed physical and psychological performance including dynamometry, 6-min walk tests and standardized questionnaires. As expected the results showed that advanced lung cancer patients experience muscular weakness, especially in the lower extremities, lower endurance performance and decreased quality of life compared to a healthy reference group [24].

In a subsequent feasibility study (POSITIVE study, Part II) our team observed favorable effects of physical exercise on the psychosocial performance and physical status of patients with advanced NSCLC that are now evaluated in the here described randomized controlled trial [24].

In addition to the benefits of physical training for quality of life, physical capacity and fatigue there is accumulating evidence that exercise can also modulate the immune system [25–36]. It is well known that the balance of specific effector T cells and immune suppressive regulatory T cells is essential for a healthy immune system and their imbalance can result of autoimmune diseases [37]. Thus, the interplay of established tumor specific effector and regulatory T cell populations might impact tumor progression, response to chemotherapy and patient prognosis [38–40]. Yet, there is little known about the effects of exercise on tumor specific adaptive immune modulation. Therefore, the scope of Positive III also includes the analysis of effects of exercise on biomarkers of immune function in cancer patients.

In conclusion, exercise provides beneficial effects in cancer patients overall [12, 41, 42]. Furthermore, there is strong evidence for physical exercise reducing cancer-related fatigue [43] and some evidence from a limited number of studies that exercise has beneficial effects on quality of life in advanced lung cancer patients. Possible pathways of how exercise may influence relevant lung cancer outcomes have not yet been studied. Therefore, the POSITIVE study (Part III) aims to investigate the benefits of a 24-week exercise intervention program in a randomized controlled setting. A translational program on immunological pathways will evaluate the potential relation between exercise-driven immunological changes and the influence on tumor specific T cell response. In contrast to other published studies, the POSITIVE study (Part III) is designed to isolate the specific impact of a 24-week exercise intervention on quality of life beyond social contact by providing social support to each study participant via weekly care management phone calls (CMPC).

## Methods and Design

### Study design

The POSITIVE study (Part III) is a randomized, controlled exercise intervention trial including patients with histologically confirmed NSCLC (stage IIIa, IIIb or IV) or SCLC (LD-SCLC, ED-SCLC), not amenable to surgery. Enrolment of patients started in November 2013, with an estimated recruitment period of approximately two and a half years. Inclusion and exclusion criteria are presented in Table 1.

**Table 1:** Inclusion and exclusion criteria. Inclusion and exclusion criteria of the POSITIVE study (Part III)

Inclusion criteria	Exclusion criteria
• NSCLC, histologically confirmed stage IIIB/IV • Receiving systemic, palliative treatment • Age ≥18 years • BMI >18 kg/m^2^ • ECOG performance status ≤2 • Signed informed consent	• Bone metastasis inducing skeletal fragility • Serious active infection • Inability to walk • Immobility (more than two days) • Previously untreated (non-irradiated or non-resected) symptomatic brain metastases • Severe neurologic or cardiac impairment • Severe respiratory insufficiency • Uncontrolled pain • Abuse of alcohol or drugs reducing compliance to the study • Any circumstance that would impede ability to give informed consent or adherence to study requirements

**Fig. 1:**
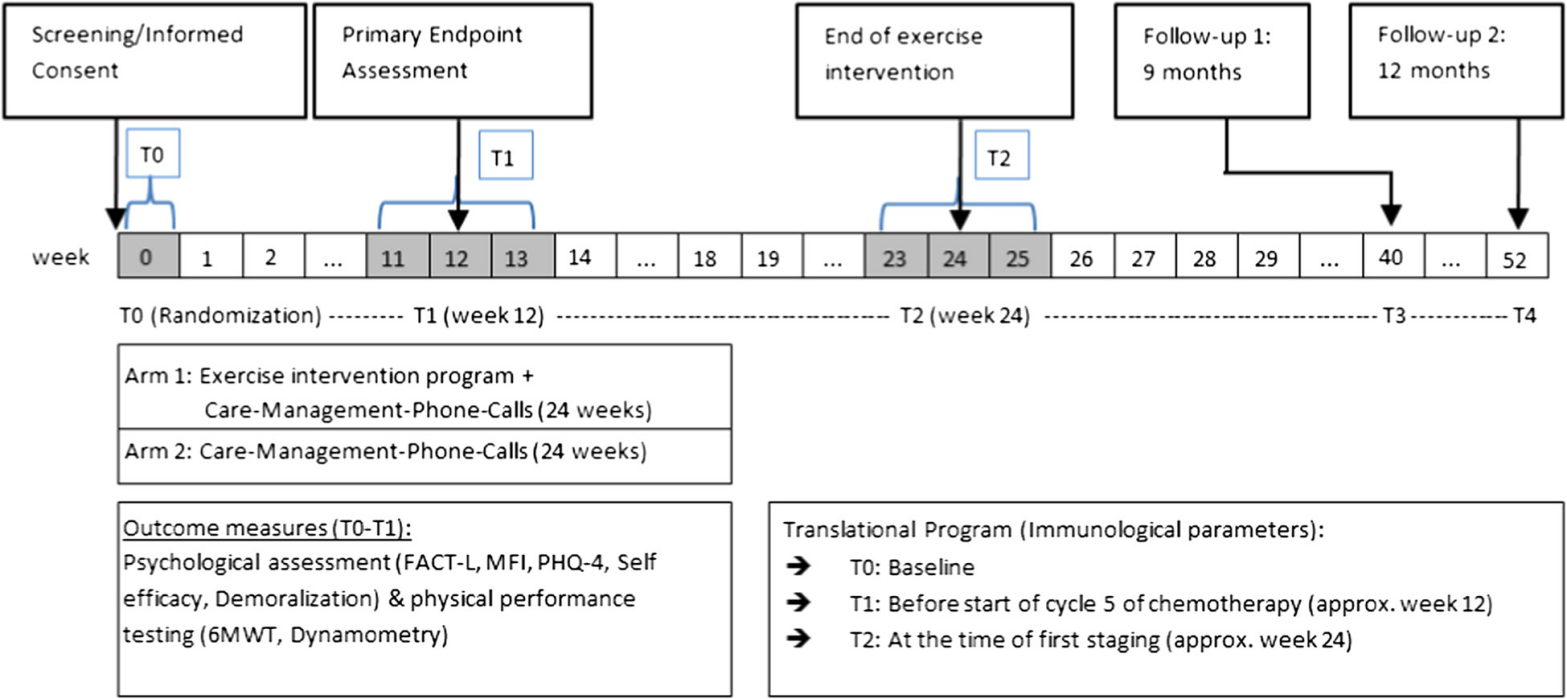
Study design. Study design of the POSITIVE study (Part III)

The study will enroll 250 patients (*n* = 125 in each arm) (Study design, Fig. 1). The first arm encompasses an “Exercise Intervention Program (EIP) and Care Management Phone Calls (CMPC)”, whereas the second arm contains “Care Management Phone Calls (CMPC)” alone. Outcome measures are assessed at baseline (T0), after 12 weeks (T1), after 24 weeks (T2) and thereafter at 9 months (T3) and 12 months (T4). The maximum study duration per patient is 1 year. The study protocol for the POSITIVE study (Part III) has been reviewed and approved by the ethics committee of the Medical Faculty of Heidelberg (S-326/2013) in November 2013.

All potential study participants at the Clinic for Thoracic Diseases Heidelberg are screened for eligibility during tumor board sessions by physicians and study personnel. Eligible patients are contacted by the study physicians; patients who provide written informed consent are scheduled for baseline assessment (T0). Randomization (block randomization, 1:1 ratio, random block sizes) to the study arms is carried out on the basis of prepared randomization lists stratified by primary treatment with radiotherapy (yes/no), histology (NSCLC/SCLC), the presence of brain metastases (yes/no) and sex (male/female).

### Objectives

Primary endpoints are (1) quality of life as measured by the FACT-L questionnaire, subcategory Physical Well-Being and (2) general fatigue measured by the Multidimensional Fatigue Inventory (MFI-20). As secondary endpoints, we evaluate whether the intervention has a positive impact on physical performance, depression, anxiety, demoralization and immunological parameters. In addition, sustainability and long-term effects of the intervention will be analyzed during the follow-up period.

### Interventions

For patients with advanced lung cancer the adequate management of disease- and treatment-related side effects determines quality of life, psychosocial burden and the extent of fatigue. Therefore weekly phone calls are provided to all patients. Care management phone calls (CMPC) are based on the Edmonton Symptom Assessment Scale (ESAS), a 9-item patient-rated symptom visual analogue scale developed for patients receiving palliative care. The modified version is comprised of one additional question to assess the patients’ quality of life. Patients rate their symptoms on an 11-point scale from 0 to 10 and at pre-specified cut off values (e.g. uncontrolled pain or breathlessness) the attending physician is contacted by the study nurse to manage symptoms and side-effects.

The exercise intervention program (EIP) contains resistance and endurance training in supervised and non-supervised sessions. Supervised training sessions are performed during patients’ inpatient stay at the clinic or in the outpatient setting near patients’ hometown in cooperating training centers within a regional network called ‘OnkoAktiv’. Non-supervised training sessions are conducted home-based. For that purpose patients receive an exercise manual and are instructed by a physiotherapist or sports scientist in individualized home-based exercises.

Exercise training is scheduled 3 times a week. The intensity of the endurance training sessions is 70-85 % of estimated maximum heart rate. Depending on patients’ individual endurance capacity, training starts at a duration of at least 15 min with subsequent increases to 45 min. Training duration and intensity for the home-based sessions is tailored using the RPE (Rate of Perceived Exertion) scale (Borg) and a well-established self-rating procedure from our lung cancer feasibility and allogeneic transplant study [44, 45]. Specifically, at the beginning of each training session patients are asked to complete an assessment of pain, fatigue, emotional status, and distress. This assessment is used to self-rate patients’ well-being and group them into 3 different categories, represented by the traffic light colors red, yellow and green, for tailoring the exercise intervention. Depending on subjectively rated well-being, the patient performs higher or lower intensity training. After each training session patients are asked to document their training in a training log. The supervised training sessions in the local outpatient training center include resistance exercise on machines and endurance training on an ergometer/treadmill. In the outpatient setting, patients and exercise specialists in cooperating training centers are instructed accordingly. During the first 12 weeks of the intervention program, supervised training sessions are conducted twice a week with an additional self-administered home-based training once a week. With the beginning of week 13 supervised training in cooperation training centers is reduced to 1x/week and patients are instructed to perform two home-based training sessions per week.

Supervised resistance and endurance training protocols comply with the American College of Sports Medicine (ACSM) exercise guidelines for cancer survivors [46] and with ACSM recommendations for progressive resistance training for novice weightlifters and older adults for one to three sets at a weight that can be done/lifted for 8 to 12 repetitions (approximately 60–80 % of 1-RM) [47, 48].

On the basis of the CMPCs the study nurse regularly reviews exercise adherence and identifies barriers and problems with regard to exercise. Exercise sessions are temporarily interrupted if pain, dizziness, or other contraindications (infections with body temperature ≥38 °C, impaired hematopoietic capacity) occur.

### Diagnostic procedures

All diagnostic procedures used in the POSITIVE study (Part III) are summarized in Table 2.

**Table 2:** Diagnostic outcomes. Assessments and instruments used in the POSITIVE study (Part III)

Outcomes	Instrument	T0	T1	T2	T3	T4
Primary endpoints						
• Physical well being	Functional Assessment for Cancer Therapy – Lung	x	x	x	x	x
• General fatigue	Multidimensional Fatigue Inventory	x	x	x	x	x
Secondary endpoints						
• Physical performance (muscle strength and endurance capacity)	Spirometry, Six-Minute Walk Test, hand-held dynamometry, exercise log	x	x	x	x	x
• Psychosocial parameters (depression, anxiety, demoralization)	Ultra-Brief Patient Health Questionnaire (PHQ-4), Demoralization Scale, locus of control, Mini Nutrition Assessment	x	x	x	x	x
• Immunological parameters (cellular immunity, cytokine and chemokine panels in T-lymphocyte subsets)	Quantification of immune cell populations and tumor antigen reactive effector (memory T cells), detection of tumor reactive regulatory T cells, quantification of key immune effector molecules in serum.	x	x	x		
Others						
• Care management phone calls	Edmonton Symptom Assessment Scale	weekly				

*Abbreviations*: T0: Baseline; T1: 12 weeks after randomization; T2: 24 weeks after randomization; T3: 1st follow-up at week 40 after randomization; T4: 2nd follow-up at week 52 after randomization

## Questionnaires

Validated questionnaires are used to assess emotional and physical well-being and to assess study outcomes.

To evaluate the primary objective “quality of life” in the subcategory “physical well-being” the lung specific part of the Functional assessment of Cancer Therapy-Lung (FACT-L) [49] is used. FACT-L is widely used in clinical studies and has already been applied in exercise intervention studies in lung cancer patients [50–52]. The PHQ-4 questionnaire is a short instrument to detect the extent of depression and anxiety [53], providing sufficient diagnostic accuracy for major depression. To evaluate the extent of chronic fatigue the Multidimensional Fatigue Inventory (MFI) is used. The MFI is divided into the five subscales “general fatigue”, “physical fatigue”, “mental fatigue”, “reduced motivation” and “reduced activity” [54] and is a proven tool in numerous oncological studies [55–57]. The Demoralization Scale (DS) [58] reliably detects the extent of existential distress in cancer patients [59] and describes the individual incapacity to cope effectively with stressful situations. The locus of control questionnaire [60, 61] measures the self-efficacy in terms of illness and health and was adapted to oncological patients to analyze perceptions of control in cancer patients [61].

The nutritional status of the patients is assessed with the Mini Nutritional Assessment (MNA). The screening part of the questionnaire allows the detection of malnutrition [62].

## Pulmonary function tests

Pulmonary function tests (PFT) are conducted to assess vital capacity (VC) and Forced Expiratory Pressure in one second (FEV1). These tests are performed during staging appointment at the Clinic for Thoracic Diseases Heidelberg.

## Physical function tests

Functional capacity is assessed by the 6-min walk test (6MWT). The 6MWT [63] is a feasible and safe test to determine patients physical functioning and is well established in cancer patients [64]. Patients are instructed to walk over a pre-measured distance of 30 m in six minutes (6-min walk distance, 6MWD) as many meters as possible. Oxygen saturation and pulse rate are monitored concomitantly before, during and after testing. After the test, patients are asked to rate their individual perceived exertion using the Borg Scale [44].

The hand-held dynamometry measures maximal voluntary isometric contraction in Newton meters in various muscle groups (device from CITEC©, Netherlands). It is a valid and reliable instrument to measure muscular strength [65]. In this study, for each test session six major muscle groups including upper and lower extremities (knee and elbow flexors and extensors, hip flexors and abductors) are assessed bilaterally for their isometric power. For each muscle group the patient performs three sets of which the best value is used in the analysis.

## Exercise log

After each training session patients randomized to the exercise training group are asked to complete an exercise log according to the FITT (Frequency, Intensity, Time and Type of exercise) criteria. Furthermore, patients are asked to report mood expressions prior and after the training sessions as well as problems or symptoms with regard to the exercise program. The perceived exertion of each exercise session is rated with the Borg Scale [44].

### Translational Program

For biomarkers of immune function EDTA blood and serum is collected and processed by the Immune Monitoring Unit at the German Center Research Center (DKFZ) Heidelberg and used to generate mononuclear cells (PBMC, cryopreserved until analysis). Using flow cytometry the question will be addressed whether exercise skews the immune response towards a pro-inflammatory phenotype by reducing the regulatory T cell frequencies. Quantification of tumor antigen reactive effector T cells will be done by IFN-γ Elispot assays from cryopreserved peripheral blood monocytes (PBMC) using a panel of defined tumor-associated antigens (TAA). These include MUC1, Her-2/neu, EGFR, telomerase and survivin [66]. Key immune effector cytokines and chemokines will be quantified through luminex technology from cryopreserved serum samples.

### Sample size

Power calculations were based on statistical tests for the group effect in the analysis of covariance for the two endpoints FACT-L-PWB and MFI tested in step 1 of the multiple testing strategy (see section Statistical Analysis). The total planned sample size is *N* = 250 (125 per arm). Patients dropping out before week 24 are not replaced. Assuming an accrual rate of about 8 patients per month the projected accrual time is approximately 2.5 years.

Power calculations were done using computer simulations written in R code. We assumed normally distributed variables with identical standard deviations at T0 and T1 weeks and an identical exponential correlation structure in both groups. Standard deviations and correlations were estimated from the pilot study. With *n* = 75 evaluable patients per group the power to detect an effect size (e) of 0.4 is 72.0 % and 80.9 % for MFI and PWB (resp.); for e = 0.45 the power is >80 % for each end point separately (5000 simulation runs per scenario). This implies that for an effect size of e ≥ 0.4 - corresponding to an estimated absolute difference (between the mean values measured at T0 and T1) of 1.35 in case of MFI and 1.6 for the PWB -, the power to detect at least one true effect if both alternative hypotheses are true is >80 %. A sample size of ≥75 evaluable patients per group corresponds to a drop-out rate of ≤ 40 %, which appears realistic. Thus, assuming an estimated 1-year survival rate of 40 % and survival times following an exponential distribution (resulting in a median survival of about 9 months), the 12-week death rate is 19.1 %.

### Data analysis

The statistical analysis of the primary and secondary quality of life (QoL) endpoints will be based on all randomized patients for whom T0 and T1 measurement are available. The main analysis of the primary and secondary QoL endpoints will use an analysis of covariance with the following regression equation:

$$ \mathbf{Q}\left(\mathbf{T}\mathbf{1}\right) = \mathbf{constant} + \mathbf{a} \times \mathbf{Q}\left(\mathbf{T}\mathbf{0}\right) + \mathbf{b} \times \mathbf{intervention}\ \mathbf{group}, $$

where Q(t) denotes the value of the QoL endpoint measured at timepoint t. The statistical testing for the group effect will be done for each primary endpoint separately. The multiple test procedure will be as follows: in a first step, the group effect for FACT-L PWB and MFI will be tested using a Bonferroni adjustment (i.e. at nominal α = 2.5 %). In a second step, FACT-L PWB and FACT-L Total will be tested using a hierarchical ordering of the hypotheses; i.e., the test regarding FACT-L Total will only be performed (again at the α = 2.5 % level) if the test for FACT-L PWB yields a significant result at the 2.5 % level. This testing strategy comprising three hypotheses controls the multiple (family-wise) level of significance α = 5 %. It is anticipated that quality of life and physical performance data will be available for most patients who are alive at week 12.

## Discussion

The aim of the POSITIVE study (Part III) is to evaluate the benefits of a 24-week exercise intervention for advanced lung cancer patients in a randomized controlled setting on quality of life and fatigue. Based on our preliminary findings, we expect a significant improvement in quality of life scores and fatigue levels after 12 weeks in the intervention compared to the control group. In addition, intervention effects on physical functioning, psychosocial parameters (e.g. depression, demoralization) and changes in immune parameters will be evaluated.

The individualized physical exercise program applied in this study is based on our previous feasibility study of an 8-week exercise intervention trial in patients with advanced NSCLC [11]. Our intervention is comprised of an endurance and resistance training that is considered to be the most frequently performed training with lung cancer patients [11, 19, 22, 23, 67–71]. To date, several studies have shown that physical exercise leads to increased physical performance in lung cancer patients undergoing surgery in the pre- and post-operative setting [16–18] and that physical exercise is safe before and after cancer treatment [72]. Yet, there is limited evidence for beneficial effects of physical activity in non-operable advanced lung cancer patients undergoing palliative treatment. Specifically the question whether an individualized endurance and resistance training is feasible for this patient population is addressed here in a large randomized setting.

Adamsen et al. (2011) reported that advanced lung cancer patients experienced physical, functional and emotional benefits after a 6-week intervention of supervised and unsupervised, home-based exercise [19]. Similar results have been reported by Quist et al. (2011) in a single-arm intervention trial. Patients conducted group exercise and individual home-based exercise for 6 weeks. Significant improvements in health-related quality of life and an increase in endurance were observed [22]. In another recently published single-arm study by Quist et al. (2015) patients with advanced stage lung cancer improved their physical capacity after a 6-week supervised group exercise intervention. With regards to psychosocial parameters specifically anxiety and emotional well-being improved, but not overall health-related quality of life (HRQoL).

Other currently ongoing studies that focus on the effects of exercise in advanced lung cancer patients might also help to generate important information about the most feasible way to improve quality of life and physical performance in this patient population [67, 70].

The ongoing POSITIVE study (Part III) enrolls only advanced, non-operable patients. The major difference to previous and ongoing studies is the inclusion of the CMPCs in the control arm. The American Society of Clinical Oncology Statement recommends an individualized care for patients with advanced cancer [73] because physical discomfort is recognized earlier and treatment could be adapted. The phone calls offer the possibility of a continuous monitoring and continuous social contact. Previous studies with metastatic cancer patients did not offer supportive strategies to this extent. Therefore, the CMPCs are an important characteristic of the POSITIVE study (Part III). Patients are called and interviewed weekly via a standardized questionnaire. On the basis of the weekly phone calls symptoms and discomfort are recognized earlier and can be managed properly. The weekly phone calls might also improve the psychosocial well-being of the patients in general [74]. These potential contacts could improve awareness to symptoms and side effects as well as facilitate coping strategies. The phone calls are provided to all patients in the study to isolate the actual effect of the exercise intervention on this dimension.

Secondary endpoints of this trial include the physical performance status of advanced lung cancer patients. Therefore, physical functioning is tested for both endurance (6MWD) and resistance (hand-held dynamometry) performance. In our study, we use the 6MWD to quantify endurance levels of the patients as it is considered as reliable predictor of survival [75, 76]. According to previous findings, an improvement of 50 meters in the 6-min walk distance induces an improved survival of 13 % [75]. A different predictor of endurance performance in lung cancer patients is aerobic capacity, assessed via VO_2_peak. This alternative approach to the 6-min walk distance has recently been proposed. Beyond beneficial effects on quality of life, physical exercise is recommended especially for lung cancer patients in order to increase VO_2_peak [77]. As described in the LUNGEVITY study VO_2_peak is becoming increasingly recognized as an outcome of major importance in NSCLC [77]. Several studies have described that VO_2_peak was not only affected by endurance training alone [78, 79] but elderly people or severely deconditioned adults can increase their absorption of oxygen with resistance training. A regain in strength in the accessory respiratory musculature also influences VO_2_peak and therewith physical performance overall. Furthermore, physical exercise has beneficial effects on immune functions. Exercise performed with resistance bands may help attenuate declines in white blood cells in lung cancer patients receiving curative intent chemotherapy [80]. It has also been observed that physical activity shows effects on, e.g. Natural Killer cytotoxic activity, lymphocyte proliferation and number of granulocytes. There were no changes observed in number of leukocytes, lymphocytes, Natural Killer cells, T lymphocytes, and pro- and anti-inflammatory mediators [42]. However, no decline in the specific immune parameters has been reported. Recommendations refer to the increasingly important role of physical exercise in cancer patients due to the ability to modulate immunity and inflammation [81]. In this context, we expect to observe beneficial effects on immune functions caused by physical activity both after 12 weeks and after 24 weeks.

## Conclusion

Lung cancer patients experience multidimensional impairments and often suffer from a large number of comorbidities affecting quality of life. In general, beneficial effects of physical exercise on fatigue levels and quality of life scores have been described [12, 82]. Prior studies have been largely performed in the pre- or post-surgical setting [14–18]. However, studies investigating the effects of physical exercise in the palliative care setting are rare. As a randomized, controlled intervention trial the POSITIVE study (Part III) will add to current knowledge about the potential benefits of exercise in quality of life in advanced lung cancer patients. In general, this study investigates the effects of a 24 week exercise intervention on (1) quality of life, (2) physical performance, (3) psychosocial and (4) immunological parameters, and (5) overall survival. An additional aim includes (6) sustainability and long-term effects. Clinical parameters will offer new insights in beneficial effects on immune functions caused by physical exercise. Overall, the focus of this investigation is on accompanying and helping patients to maintain independent function as long as possible. If our intervention has measurable benefits for lung cancer patients, it can be translated to and implemented in the clinical setting.

## Abbreviations

6MWD, 6-minute walk distance; 6MWT, 6-minute walk test; ACSM, American college of sports medicine; BMI, body mass index; CMPC, care management phone call(s); DKFZ, German cancer research center; DS, demoralization scale; ECOG, Eastern cooperative oncology group; ED-SCLC, extensive disease, small cell lung cancer; EIP, exercise intervention program; ESAS, Edmonton symptom assessment scale; FACT-L, Functional assessment of cancer therapy – Lung; FEV1, forced expiratory pressure in one second; HRQoL, health-related quality of life; LD-SCLC, limited disease, small cell lung cancer; MFI, multidimensional fatigue inventory; MNA, mini nutrition assessment; NSCLC, non-small cell lung cancer; PBMC, peripheral blood mononuclear cells; PFT, pulmonary function tests; PHQ, physical health questionnaire; PWB, physical well-being; QoL, quality of Life; SCLC, small cell lung cancer; TAA, tumor-associated antigens; VC, vital capacity
